# Editorial: The Analysis of Nanovesicles, Biomaterials and Chemical Compounds: Assisting the Promotion of Angiogenesis and Enhancing Tissue Engineering Strategies

**DOI:** 10.3389/fcvm.2022.904738

**Published:** 2022-04-26

**Authors:** Vincenzo Lionetti, Niketa Sareen, Sanjiv Dhingra

**Affiliations:** ^1^Unit of Translational Critical Care Medicine, Institute of Life Sciences, Scuola Superiore Sant'Anna, Pisa, Italy; ^2^Regenerative Medicine Program, Department of Physiology and Pathophysiology, Institute of Cardiovascular Sciences, St. Boniface Hospital Albrechtsen Research Centre, University of Manitoba, Winnipeg, MB, Canada

**Keywords:** cardiovascular disease, stem cells, cardiac repair, extracellular veiscles, nanovesicles, nanomaterials, tissue engineering

Heart disease remains the leading cause of morbidity and mortality worldwide. The loss of functional cardiomyocytes after an ischemic or non-ischemic injury leads to permanent damage to the heart muscle as cardiomyocytes are terminally differentiated and are not able to replace the damaged cells. The currently available pharmacological therapies are able to provide temporary relief and improve life span of cardiac patients. However, these interventions are not able to offer permanent solution to the problem. Thus, there is an urgent need for new and more effective approaches that can provide permanent treatment options for the heart disease. In this regard stem cell-based therapies have emerged as promising solution (Figure 1) (1, 2). Stem cells have the ability to differentiate into cardiomyocytes and replenish lost cardiac cells due to cardiac injury. In fact in different experimental animal models of heart failure, transplantation of stem cells led to improvement in cardiac function and the mechanisms of cardiac repair were dependent upon the type of stem cells employed (3). Some pre-clinical studies suggested that pluripotent cells such as embryonic stem cells and induced pluripotent stem cells (iPSC) promote cardiac repair by differentiating into beating cardiomyocytes in the heart after implantation (4, 5). On the other hand, bone marrow derived and adipose tissue derived mesenchymal stem cells (MSCs) are reported to promote cardiac repair through paracrine mechanisms (1, 6). MSCs are known to secrete several growth factors, angiogenic, antiapoptotic factors and immunosuppressive molecules. These paracrine factors are responsible for cardiac repair by promoting angiogenesis in the infarcted heart, limiting cardiomyocyte loss, and suppressing inflammation caused by immune cell infiltration. Although the therapeutic efficacy of MSCs has been clearly demonstrated in different animal models and no side effects were observed in patients after transplantation of MSCs in the heart, only very few phase III clinical trials using MSCs have shown encouraging results (7). Indeed, beneficial effects of transplanted MSCs in terms of cardiac repair were very short lived, and major reason for this poor performance of MSCs was limited survival of transplanted cells in the injured heart. Therefore, the strategies that can improve the survival of transplanted stem cells in the heart could be of immense therapeutic value. In this regard, investigators working in the field have suggested different approaches to improve survival of implanted MSCs in the heart (8). Some of the studies reported that preconditioning of MSCs with chemical compounds such as hyaluronan mixed ester of butyric and retinoic acid (9, 10) and overexpression of different cell survival proteins such as AKT and ERK ½ can improve survival of implanted cells in the heart (11). Several other studies reported that microRNA (miRNA) mediated approaches are also able to promote survival of transplanted cells. Role of multiple miRNAs in survival of MSCs has been studied and reported by different investigators (12). Some of the miRNAs which have been reported to promote MSCs survival include miR-1, miR-210, miR-378, miR-34a, miR-21, miR-23a, miR-503 (13). One such study in this special issue highlights the role of miR-377 in mediating cardiomyocyte death. In this study Henderson et al. evaluated the effect of miR-377 on DOX induced cardiotoxicity in mouse model and reported that inhibiting miR-377 results in changes in pathways regulating cell proliferation, inflammation, apoptosis and differentiation.

**Figure 1:**
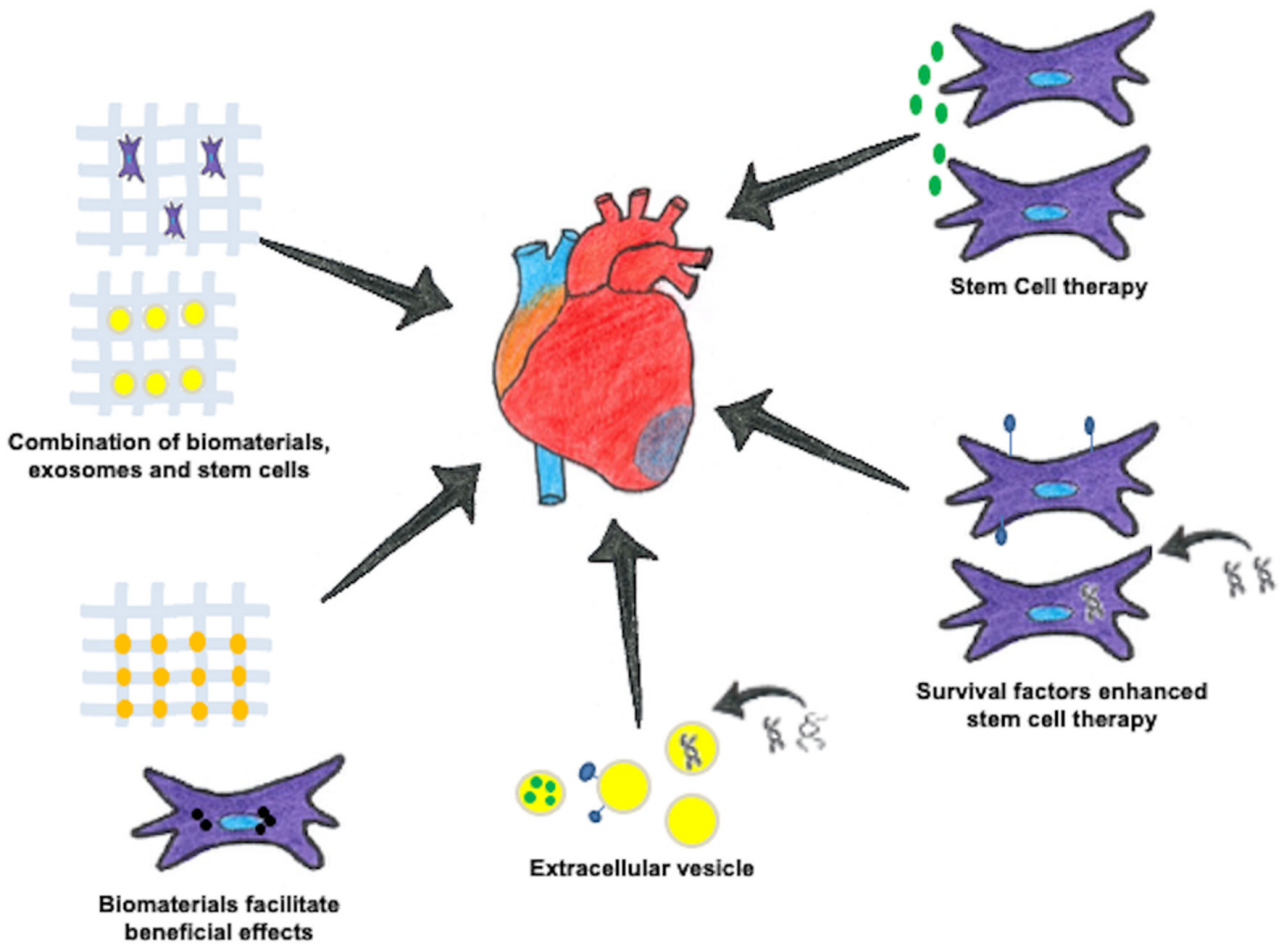
Different approaches for cardiac regeneration. Cell therapy using stem cells can help treat the damaged myocardium. These stem cells enriched with different biomolecules can exhibit positive effects on the heart muscle. The extracellular vesicles released from stem cells have also emerged as excellent source of therapeutic agents. Using specially engineered biomaterials aids in creating a 3D microenvironment which can be injected alone or in combination with stem cells.

Recently, biomaterials based approaches are also being explored to improve survival of transplanted stem cells in the heart. In this regard, hydrogels and nanoparticles mediated delivery of stem cells has shown significant promise (Figure 1). In past, some of the common natural and synthetic hydrogels used in MSC therapy include collagen, alginate, hyaluronic acid, chitosan or poly glycerol sebacate derivatives (14). The hydrogels and nanoparticles can also be tagged with soluble factors such as prostaglandin E_2_ (PGE_2_) and fibroblast growth factor (FGF) that promote survival of stem cells in the myocardium (1, 15). In addition to this, these biomaterials can be used as a delivery vehicle for transporting drugs or biochemical agents to the damaged myocardium for beneficial effects. Alvi et al., discuss this aspect of nanoparticles, microspheres and hydrogels in modulating positive effects by employing different fabrication and formulation strategies. They also highlight the clinical trials being carried out using this set of biomaterials for improving cardiac condition. Furthermore, hydrogels made of extra cellular matrix (ECM) based biomaterials can act as bulking agents and are also reported to prevent inflammatory response in heart due to their ECM like structure (16).

The nanoparticles also offer several advantages due to their physical properties including size, charge, shape, surface area and functional groups (17, 18). The metal-based nanoparticles, quantum dots and magnetic nanoparticles are most commonly used for stem cells and drug delivery. Gokce et al., review the applications of currently used 2D materials for cardiac regeneration. Their report provides a deeper understanding of these biomaterials in terms of their immunomodulatory properties and inducing cardioprotective effects. Additionally, biomaterials are also reported to aid in tracking of transplanted stem cells due to their inherent fluorescence abilities (19).

In addition to stem cells as source of cell therapy, in some of the recent studies, stem and progenitor cells derived extracellular vesicles (EVs) or exosomes have garnered a lot of attention by acting as cell free systems for cardiac repair following myocardial injury (20). A detailed review on the role of stem cells derived exosomes has been compiled by Jayaraman et al. in this special issue. The authors have provided insights about different aspects of exosome biogenesis, storage and preservation with emphasis on their role in mediating the positive effects in cardiac ailments. The study highlights major advantages of using exosomes over stem cells for cardiac therapy; along with some of the key points to consider before large scale application of exosomes involving isolation and storage. The EVs are reported to exhibit beneficial effects by acting as reservoirs of paracrine factors that are produced by MSCs and progenitor cells. In addition to this, EVs are also reported to act as cargos to deliver important biomolecules such as microRNAs, metabolites, amino acids, lipids, DNA, proteins or even drugs to target tissues (21, 22). Pant et al., discuss the therapeutic role of non-coding RNAs (ncRNA) in exosomes. This review covers the role of ncRNA contained in MSC derived exosomes in cardiac, bone and cartilage tissue regeneration. They have also highlighted miRNA mediated applications of exosomes in neurodegenerative disorders as well as in cancer. Based on the type of cells EVs are derived from, these vesicles can differ in their contents, thus, resulting in different effects at the injection site. This phenomenon is demonstrated in research conducted by Ranjan et al., where exosomes sourced from myofibroblasts were responsible for induction of apoptosis and reduced proliferation of endothelial cells. Moreover, these exosome treated endothelial cells also exhibit reduced angiogenesis and cell migration.

In addition to this, conventional cardiovascular drugs such as ticagrelor may modulate the release of exosomes from cardiac progenitor cells (23). Multiple reports have also suggested that EVs or exosomes promote cardiac repair by preventing apoptosis, inflammation and oxidative stress, as well as by improving angiogenesis and cardiac remodeling (Jayaraman et al.) (24, 25). These aspects of EV mediated cardioprotection have been discussed in this special issue by Liu et al.. The review covers the role of EVs derived from multiple sources in treating cardiac pathologies. The authors also discuss various strategies to modify EVs for better therapeutic effects. However, besides preventing negative remodeling, EVs can also act as important markers of cardiac pathology by acting as inter cell communicators of immune cells. This aspect of EVs has been elaborated in the article by Jadli et al.. The authors discuss important aspect of EV mediated signaling in cardiac pathologies like atherosclerosis, HF, ischemic heart disease and various forms of cardiomyopathies.

Some of the recent studies have suggested that delivery of EVs using biomaterials prevents their wash out at the site of injection (26). Furthermore, the application of a combination of biomaterials and exosomes is also reported to suppress elevated immune response, promote tissue regeneration and wound healing (27). Therefore, a combination of different approaches may be a better approach for future regenerative strategies for cardiac repair (26). However, there is a need to conduct more extensive studies in clinically relevant animal models of heart failure using these new approaches.

The goal of this focused issue was to bring together state-of-the-art original research and review articles from experts in the field of cardiovascular disease, stem cells, nanomaterials, extracellular nanovesicles and microRNAs, with special focus on translational aspects of these approaches. Our hope is that in future the readers would benefit from the articles in this special issue and come up with more effective state-of-the-art tools to repair injured myocardium in patients.

## Author Contributions

The study was conceptualized and designed by VL and SD. VL, NS, and SD carried out literature search, and prepared manuscript and figure. All authors have read and approved the final manuscript.

## Funding

This work was supported by funding from the Canadian Institutes of Health Research to SD and by funding from Fondazione Pisa (Italy; Etherna: prog. n. 161/16) to VL. NS was supported by Go for IT Project of Fondazione CRUI (Italy; CardioNEX).

## Conflict of Interest

The authors declare that the research was conducted in the absence of any commercial or financial relationships that could be construed as a potential conflict of interest.

## Publisher's Note

All claims expressed in this article are solely those of the authors and do not necessarily represent those of their affiliated organizations, or those of the publisher, the editors and the reviewers. Any product that may be evaluated in this article, or claim that may be made by its manufacturer, is not guaranteed or endorsed by the publisher.
